# Association of Air Pollution Exposure in Childhood and Adolescence With Psychopathology at the Transition to Adulthood

**DOI:** 10.1001/jamanetworkopen.2021.7508

**Published:** 2021-04-28

**Authors:** Aaron Reuben, Louise Arseneault, Andrew Beddows, Sean D. Beevers, Terrie E. Moffitt, Antony Ambler, Rachel M. Latham, Joanne B. Newbury, Candice L. Odgers, Jonathan D. Schaefer, Helen L. Fisher

**Affiliations:** 1 Department of Psychology and Neuroscience, Duke University, Durham, North Carolina; 2 King’s College London, Social, Genetic, and Developmental Psychiatry Centre, Institute of Psychiatry, Psychology, and Neuroscience, London, United Kingdom; 3 Economic and Social Research Council Centre for Society and Mental Health, King’s College London, London, United Kingdom; 4 Environmental Research Group, School of Public Health, Imperial College London, London, United Kingdom; 5 Medical Research Council Centre for Environment and Health, Imperial College London, United Kingdom; 6 Center for Genomic and Computational Biology, Duke University, Durham, North Carolina; 7 Department of Psychiatry and Behavioral Sciences, Duke University, Durham, North Carolina; 8 PROMENTA, Department of Psychology, University of Oslo, Oslo, Norway; 9 Bristol Medical School: Population and Health Sciences, University of Bristol, Bristol, United Kingdom; 10 Department of Psychological Science, University of California, Irvine; 11 Social Science Research Institute, Duke University, Durham, North Carolina; 12 Institute of Child Development, University of Minnesota, Minneapolis

## Abstract

**Importance:**

Air pollution exposure damages the brain, but its associations with the development of psychopathology are not fully characterized.

**Objective:**

To assess whether air pollution exposure in childhood and adolescence is associated with greater psychopathology at 18 years of age.

**Design, Setting, and Participants:**

The Environmental-Risk Longitudinal Twin Study is a population-based cohort study of 2232 children born from January 1, 1994, to December 4, 1995, across England and Wales and followed up to 18 years of age. Pollution data generation was completed on April 22, 2020; data were analyzed from April 27 to July 31, 2020.

**Exposures:**

High-resolution annualized estimates of outdoor nitrogen oxides (NO_x_) and particulate matter with aerodynamic diameter less than 2.5 μm (PM_2.5_) linked to home addresses at the ages of 10 and 18 years and then averaged.

**Main Outcomes and Measures:**

Mental health disorder symptoms assessed through structured interview at 18 years of age and transformed through confirmatory factor analysis into continuous measures of general psychopathology (primary outcome) and internalizing, externalizing, and thought disorder symptoms (secondary outcomes) standardized to a mean (SD) of 100 (15). Hypotheses were formulated after data collection, and analyses were preregistered.

**Results:**

A total of 2039 participants (1070 [52.5%] female) had full data available. After adjustment for family and individual factors, each interquartile range increment increase in NO_x_ exposure was associated with a 1.40-point increase (95% CI, 0.41-2.38; *P* = .005) in general psychopathology. There was no association between continuously measured PM_2.5_ and general psychopathology (b = 0.45; 95% CI, −0.26 to 1.11; *P* = .22); however, those in the highest quartile of PM_2.5_ exposure scored 2.04 points higher (95% CI, 0.36-3.72; *P* = .02) than those in the bottom 3 quartiles. Copollutant models, including both NO_x_ and PM_2.5_, implicated NO_x_ alone in these significant findings. NO_x_ exposure was associated with all secondary outcomes, although associations were weakest for internalizing (adjusted b = 1.07; 95% CI, 0.10-2.04; *P* = .03), medium for externalizing (adjusted b = 1.42; 95% CI, 0.53-2.31; *P* = .002), and strongest for thought disorder symptoms (adjusted b = 1.54; 95% CI, 0.50-2.57; *P* = .004). Despite NO_x_ concentrations being highest in neighborhoods with worse physical, social, and economic conditions, adjusting estimates for neighborhood characteristics did not change the results.

**Conclusions and Relevance:**

Youths exposed to higher levels of outdoor NO_x_ experienced greater psychopathology at the transition to adulthood. Air pollution may be a nonspecific risk factor for the development of psychopathology.

## Key Points

**Question:**

Is exposure to outdoor air pollution in childhood and adolescence associated with the development of psychopathology at the transition to adulthood?

**Findings:**

In this cohort study of 2039 UK-born children followed up for 2 decades, early-life exposure to nitrogen oxides was significantly associated with general psychopathology at 18 years of age, representing greater internalizing, externalizing, and thought disorder symptoms. The associations were not attributable to individual or family-level factors or to disadvantageous neighborhood characteristics.

**Meaning:**

These findings suggest that exposure to nitrogen oxides in early life may be a nonspecific risk factor for the development of psychopathology as young people begin the transition to adulthood.

## Teaser

This cohort study examines the association between exposure to outdoor air pollution in childhood and adolescence with the development of psychopathology at the transition to adulthood.

## Introduction

Outdoor air pollution is a toxicant mix with known cardiovascular and respiratory health effects.^1^ Emerging evidence suggests that air pollution can also directly (eg, via translocation of ultrafine-pollutant particles across the nasal olfactory nerve) or indirectly (eg, via inflammatory signaling from other organ systems, particularly the lungs) harm the central nervous system (CNS).^2,3^ Air pollution exposure has consequently been implicated in diverse CNS damage, including vascular damage, chronic neuroinflammation, glial-cell dysregulation, and, in children, global impairments to brain structural integrity, neuron proliferation, and signaling cascades.^4,5,6^

Although the cellular effects of air pollution exposure are well described, the functional consequences have yet to be fully characterized. In particular, emerging observational evidence has implicated air pollutants in risk for varied psychiatric disorders, including attention-deficit/hyperactivity disorder, depression, anxiety, and schizophrenia.^7,8,9,10,11,12,13^

This evidence base has limitations. First, previous work has been based on cross-sectional observations, limiting causal inference. Second, most work has primarily examined exposure and mental illness among adults, making it difficult to determine what role pollution may have played in the development of psychiatric symptoms, which tend to first emerge in childhood and adolescence.^14,15^ Child air pollution exposure is a particular concern because neurodevelopment extends from the prenatal period through adolescence,^16^ with maturational processes (eg, cortical thinning) continuing into young adulthood.^17^ Immature cerebral vasculature may also render children more vulnerable to toxicants.^18^ Third, previous work has generally examined associations between air pollution and individual psychiatric disorders, an approach that does not take into account the dimensional nature of most psychiatric problems or the high rates of concurrent and sequential comorbidity among disorders.^14^ Fourth, few studies have been able to rule out key threats to causal inference posed by the potential self-selection into poor-air neighborhoods by families with greater liability to mental illness or the potential influence of disadvantageous aspects of the neighborhood environment associated with air pollutant concentrations and mental illness (eg, poverty, crime, and physical dilapidation).^19,20^ Fifth, previous work has often relied on city-level or aggregate air quality data, making findings prone to the ecological fallacy.

The current study uses data from a nationally representative UK cohort followed up from birth to 18 years of age to test the hypothesis that greater air pollution exposure in childhood and adolescence is associated with greater psychopathology at the transition to adulthood (ie, 18 years of age). To address the limitations of prior research, air pollution exposure was measured using high-resolution (20 × 20 m: address level) estimates of the 2 pollutants most evaluated with respect to CNS disease,^21^ particulate matter with aerodynamic diameter less than 2.5 μm (PM_2.5_) and nitrogen oxides (NO_x_), each assessed when individuals were 10 and 18 years of age. Because the existing evidence suggests that air pollution is associated with increased risk of multiple disorders, psychopathology was measured using a continuous latent factor that captured general liability to multiple types of psychiatric problems, known as the general psychopathology p-factor (prespecified primary outcome).^22,23,24,25^ To ensure that associations with general psychopathology were not attributable to a single domain of psychiatric symptoms, air pollution associations were also examined with the 3 constituent correlated factors of the p-factor: internalizing, externalizing, and thought disorder symptoms (prespecified secondary outcomes). Finally, to rule out confounding attributable to family self-selection or disadvantageous aspects of the neighborhood environment, air pollution and psychopathology associations were adjusted using a series of high-quality measures of family and individual factors and of disadvantageous neighborhood characteristics, including socioeconomic deprivation, physical dilapidation, social disconnection, and dangerousness.

## Methods

### Sample

Participants were members of the Environmental-Risk (E-Risk) Longitudinal Twin Study, which tracks the development of a nationally representative cohort of 2232 twins born in 1994 to 1995 in England and Wales and initially assessed at 5 years of age. The full sample comprised 56% monozygotic and 44% dizygotic twin pairs; sex was evenly distributed within zygosity (49% male). Follow-up home visits were conducted when participants were 7 (98% participation), 10 (96%), 12 (96%), and 18 (93%) years of age. The cohort was evenly distributed across England and Wales (eFigure 1 in the Supplement), and the cohort’s neighborhoods represent the full range of socioeconomic conditions in Great Britain. eFigure 2 in the Supplement shows that E-Risk study families’ addresses are a near-perfect match to the deciles of the UK government’s 2015 Index of Multiple Deprivation, which ranks British neighborhoods in terms of relative deprivation at an area level of approximately 1500 residents; approximately 10% of the E-Risk study cohort fills each of the index’s 10% bands, indicating that the cohort accurately represents the distribution of deprivation in the United Kingdom. eTable 1 in the Supplement displays sociodemographic characteristics of the E-Risk study participants at 18 years of age. Further details are reported elsewhere^26^ and in eAppendix 1 in the Supplement. The Joint South London and Maudsley and the Institute of Psychiatry Research Ethics Committee approved each phase of the study. Parents gave written informed consent, and twins between 5 and 12 years of age gave assent and then written informed consent at 18 years of age. This study followed the Strengthening the Reporting of Observational Studies in Epidemiology (STROBE) reporting guidelines.^27^

### Measures

#### Air Pollution Exposure in Childhood and Adolescence

Mean annual exposure to NO_x_, a regulated gaseous pollutant composed of nitric oxide (NO) and nitrogen dioxide (NO_2_), and to PM_2.5_, a regulated aerosol pollutant with suspended solid and liquid particles less than 2.5 μm in diameter, was estimated based on the latitude-longitude coordinates of participants’ residential addresses at 10 and 18 years of age.^8^ The exposure estimation procedures have been previously described.^28^ Briefly, exposure was modeled at the local scale using emissions data from the UK National Atmospheric Emissions Inventory and the Imperial College’s UK road-traffic emissions inventory. Air quality was modeled down to individual streets, providing annual mean estimates of NO_x_ and PM_2.5_ at 20 × 20-m grid points throughout the country (ie, at address level). Participants’ long-term exposure in childhood and adolescence was estimated by taking the mean of the 2 annual exposure estimates. Estimates from the 2 ages were highly correlated (NO_x_ *r* = 0.83, *P* < .001; PM_2.5_ *r* = 0.87, *P* < .001), as were the resulting long-term mean estimates of the 2 pollutants (*r* = 0.83, *P* < .001). Pollution data generation was completed on April 22, 2020; data were analyzed from April 27 to July 31, 2020. Further details are given in eAppendix 2 and eTable 2 in the Supplement.

#### Psychopathology at the Transition to Adulthood

##### Assessment of Symptoms of Mental Disorder

At 18 years of age, participants were interviewed about past-year symptoms of mental disorder. These methods have been previously described.^29^ Briefly, 5 externalizing-spectrum disorder symptoms were assessed, including alcohol dependence, cannabis dependence, tobacco dependence, conduct disorder, and attention-deficit/hyperactivity disorder. Four internalizing-spectrum disorder symptoms were assessed, including depression, generalized anxiety disorder, posttraumatic stress disorder, and eating disorder. Thought disorder symptoms were assessed via 7 items about delusions and hallucinations and 6 items about unusual thoughts and feelings. Details are given in eAppendix 3 in the Supplement.

##### The Structure of Psychopathology

Using confirmatory factor analysis, we estimated 2 standard models^30,31^ that are frequently used to examine hierarchically structured constructs: a correlated-factors model with 3 factors (representing internalizing, externalizing, and thought disorder) and a bifactor model specifying a general psychopathology factor (eFigure 3 in the Supplement) in addition to the 3 specific factors. Symptoms corresponding to disorders of distress (depression, generalized anxiety disorder, and posttraumatic stress disorder) and eating pathology loaded on the internalizing factor; symptoms corresponding to disorders of substance use (alcohol, cannabis, and tobacco) and oppositional behavior (conduct disorder and attention-deficit/hyperactivity disorder) loaded on the externalizing factor; and symptoms corresponding to disorders associated with psychosis loaded on the thought disorder factor. Factor loadings and model fit are provided in the article by Schaefer et al^29^ and summarized in eAppendix 3 in the Supplement. Models fit the data well. For expository purposes, scores on each factor were scaled to a mean (SD) of 100 (15).

#### Family- and Individual-Level Covariates

Family-level covariates included family socioeconomic status and family psychiatric history. Individual-level covariates included participant history of emotional and behavioral problems in early childhood and tobacco smoking. Details on these covariates are provided in eAppendix 4 in the Supplement.

#### Disadvantageous Aspects of the Neighborhood Environment

Disadvantageous neighborhood characteristics were measured through ecological risk assessment that combines information from 4 independent sources of data: (1) geodemographic data from local governments, (2) official crime data from the UK Police, (3) Google street view–based systematic social observation, and (4) surveys of neighborhood residents, conducted by the E-Risk study team. These data sources were used to measure 4 neighborhood characteristics across childhood (from 5 to 17 years of age): deprivation, dilapidation, disconnection, and dangerousness. These measures have been previously described,^32,33^ and details are given in eAppendix 5 in the Supplement. An overall composite ecological-risk index was created by summing values across the 4 measures.

### Statistical Analysis

#### Testing Air Pollution–Psychopathology Associations

The first analytic step investigated air pollution associations with psychopathology. First, associations between air pollution exposure and the primary outcome, general psychopathology, were tested using ordinary least squares multiple linear regression following 4 models regressing the outcome onto the 2 pollutants separately: (1) a sex-adjusted baseline model taking into account known sex differences in psychopathology; (2) a family factors–adjusted model adjusting for sex, family socioeconomic status, and family psychiatric history; (3) an individual factors–adjusted model adjusting for sex and participant history of emotional and behavioral problems in early childhood and tobacco smoking up to 18 years of age; and (4) a fully adjusted model, including all family and individual factors. All models were run using the air pollutants measured continuously and rescaled to interquartile range increments, which is a common approach to enable comparison of statistical effect sizes across air pollutants with different absolute concentration ranges, and dichotomized to the top quartile vs the bottom 3 quartiles, following the methods of Newbury et al,^8^ as a sensitivity test to index potential threshold effects that occur at the high end of exposures, which exceeded current health guidelines (see eTable 1 in the Supplement for the quartile cutoffs).

Second, to test for specificity in psychopathology symptom associations, the above modeling steps were performed again with the secondary outcomes, the correlated factors of internalizing, externalizing, and thought disorder symptoms.

Third, to test for specificity in individual pollutant associations, the fully adjusted model with general psychopathology was performed again with both air pollutants, NO_x_ and PM_2.5_, included simultaneously to produce a copollutant model.

Fourth, to test for specificity in developmental timing of associations, all fully adjusted models were rerun with the pollution measures decomposed into separate estimates for the ages of 10 and 18 years. Sensitivity tests performed these models again using only participants who did not change their address before 10 years of age (n = 1277 [62.6%]) or between 10 and 18 years of age (n = 1457 [71.5%]) to keep neighborhood conditions as consistent as possible.

#### Accounting for Disadvantageous Neighborhood Characteristics

The second analytic stage sought to account for disadvantageous neighborhood characteristics that may be correlated with air pollution and that could otherwise account for air pollution–psychopathology associations. Only significant air pollutants identified in the first stage were carried forward to the second. All fully adjusted models were rerun with additional adjustment for each measure of the neighborhood environment (deprivation, dilapidation, disconnection, and dangerousness) one at a time and then collectively via the overall Ecological Risk Index. Given documented associations of air pollution with urbanicity and urbanicity with mental illness,^34,35^ additional adjustment was also applied for a 3-category measure of urbanicity (urban, n = 635 [32.1%]; intermediate, n = 942 [47.7%]; and rural, n = 400 [20.2%]; eAppendix 5 in the Supplement). As with the first analytic stage, pollutant(s) were analyzed as both continuous measures and dichotomized at the top quartile to test threshold effects.

Because the E-Risk study comprises twins, the nonindependence of participants within families was accounted for in all models by adjusting the SEs. Analyses were conducted in Stata, version 16.1 (StataCorp LLC). All analyses were prespecified; study premise and analysis plan were preregistered.^36^ Findings were checked for reproducibility by an independent data analyst, who recreated the code by working from the manuscript and applied it to a fresh data set. Significance tests were 2-tailed α = .05.

## Results

A total of 2039 participants (1070 [52.5%] female) had full air pollution and psychopathology data. Attrition analysis is detailed in eAppendix 6 in the Supplement. Mean annualized NO_x_ exposure in childhood and adolescence in the analytic sample ranged from 2.45 to 113.07 μg/m^3^ (mean [SD], 29.55 [15.44]). A total of 447 participants’ (21.9%) NO_x_ exposure exceeded World Health Organization (WHO) guidelines^37^ for NO_2_ (≥40 μg/m^3^), a component of NO_x_. Mean annualized PM_2.5_ exposure in childhood and adolescence ranged from 2.92 to 19.34 μg/m^3^ (mean [SD], 11.59 [2.08]). A total of 1716 participants’ (84.2%) PM_2.5_ exposure exceeded WHO guidelines^37^ (≥10 μg/m^3^).

### Association of Air Pollution Exposure in Childhood and Adolescence With Psychopathology at the Transition to Adulthood

On average, children and adolescents exposed to higher levels of continuously measured NO_x_ air pollution had greater psychopathology at the transition to adulthood (Table 1). After sex was adjusted for, each interquartile range increment increase in NO_x_ exposure was associated with a 1.45-point increase (95% CI, 0.34-2.55; *P* = .01) in general psychopathology (on a mean [SD] scale of 100 [15]). Adjustment for family socioeconomic status, family psychiatric history, participant history of emotional and behavioral problems, and participant tobacco smoking did not change the results (fully adjusted b = 1.40; 95% CI, 0.41-2.38; *P* = .005). In prespecified sensitivity tests, associations of NO_x_ with general psychopathology remained statistically significant when air pollution was dichotomized at the top quartile of exposure (top quartile vs the bottom 3 quartiles) to test potential threshold effects at the highest levels of exposure, which exceeded WHO guidelines (Table 1). After full adjustment for family and individual factors, participants in the highest quartile of NO_x_ exposure in childhood and adolescence scored 2.62 points higher on general psychopathology than their peers in the bottom 3 quartiles (95% CI, 0.96-4.27; *P* = .002).

**Table 1.** Association of Exposure to NO_x_ and PM_2.5_ Air Pollution in Childhood and Adolescence With General Psychopathology at 18 Years of Age^a^

Model	Air pollution exposure measured continuously and scaled to the interquartile range				Air pollution exposure dichotomized to test the highest exposures (top quartile vs bottom 3 quartiles)			
	NO_x_		PM_2.5_		NO_x_		PM_2.5_	
	b (95% CI)	*P* value	b (95% CI)	*P* value	b (95% CI)	*P* value	b (95% CI)	*P* value
Sex-adjusted model	1.45 (0.34 to 2.55)	.01	0.28 (−0.52 to 1.07)	.49	2.78 (0.91 to 4.66)	.004	2.21 (0.33 to 4.08)	.02
Family factors-adjusted model^b^	1.26 (0.23 to 2.29)	.02	0.31 (−0.45 to 1.06)	.43	2.25 (0.46 to 4.04)	.01	1.82 (0.03 to 3.61)	.05
Individual factors-adjusted model^c^	1.40 (0.38 to 2.42)	.007	0.39 (−0.33 to 1.11)	.29	2.75 (1.05 to 4.45)	.002	2.15 (0.42 to 3.87)	.02
Fully adjusted model^d^	1.40 (0.41 to 2.38)	.005	0.45 (−0.26 to 1.15)	.22	2.62 (0.96 to 4.27)	.002	2.04 (0.36 to 3.72)	.02

Abbreviations: NO_x_, a regulated gaseous pollutant composed of nitric oxide and nitrogen dioxide; PM_2.5_, a regulated aerosol pollutant with suspended solid and liquid particles smaller than 2.5 μm in diameter.

^a^ The table presents analyses conducted in the full analytic sample of participants with complete air pollution and psychopathology data (analytic sample N = 2039). The b coefficients represent unit change in psychopathology factor scores per interquartile range increment increase in pollutant exposure (in the b coefficient columns under the “air pollution exposure measured continuously and scaled to the interquartile range” heading) and moving from the bottom 3 quartiles of air pollutant exposure to the top quartile (in the b coefficient columns under the “air pollution exposure dichotomized to test the highest exposures [top quartile vs bottom 3 quartiles” heading]). General psychopathology was standardized to a mean (SD) of 100 (15). The nonindependence of children within families was accounted for in all models by adjusting the SEs.

^b^ The family factors model was adjusted for sex, family socioeconomic status, and family psychiatric history.

^c^ The individual factors model was adjusted for sex, participant history of emotional and behavioral problems, and tobacco smoking.

^d^ The fully adjusted model was adjusted for sex, family socioeconomic status, family psychiatric history, participant history of emotional and behavioral problems, and tobacco smoking.

In contrast to the findings for NO_x_, exposure to continuously measured PM_2.5_ was not associated with general psychopathology, regardless of adjustment for covariates (b = 0.45; 95% CI, −0.26 to 1.15; *P* = .22) (Table 1). However, prespecified sensitivity tests of pollution at the highest levels using PM_2.5_ dichotomized at the top quartile of exposure revealed that exposure to PM_2.5_ was significantly related to general psychopathology in this analysis of extremes (Table 1). After full adjustment for family and individual factors, participants in the highest quartile of PM_2.5_ exposure scored 2.04 points higher on general psychopathology (95% CI, 0.36-3.72; *P* = .02) than their peers in the bottom 3 quartiles.

**Figure 1.**
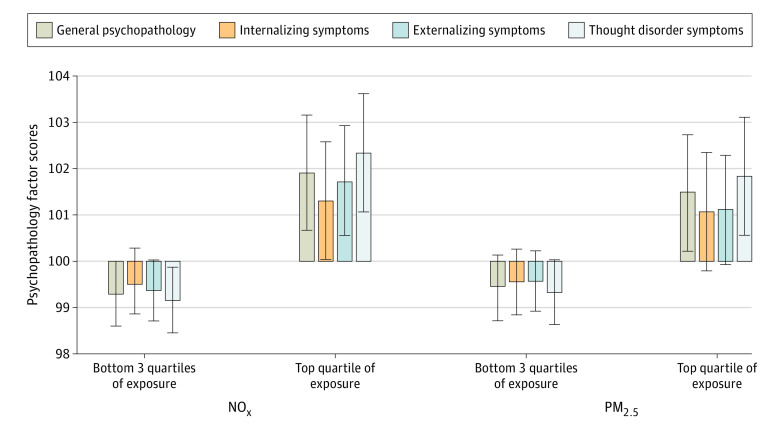
Mean Psychopathology Factor Scores at Age 18 Years for Participants at the Top vs Bottom 3 Quartiles of Nitrogen Oxides (NO_x_) and Particulate Matter Less Than 2.5 μm (PM_2.5_) Exposure in Childhood and Adolescence Mean estimates were adjusted for covariates of sex, family socioeconomic status, family psychiatric history, participant history of emotional and behavioral problems, and tobacco smoking. Error bars present 95% CIs. Psychopathology factors were scaled within the cohort to a mean (SD) of 100 (15). After full adjustment for family and individual factors, participants in the highest quartile of NO_x_ exposure in childhood and adolescence scored 2.62 points higher (95% CI, 0.96-4.27 points; *P* = .002) on general psychopathology than their peers in the bottom 3 quartiles, and participants in the highest quartile of PM_2.5_ exposure scored 2.04 points higher (95% CI, 0.36-3.72; *P* = .02).

Table 2 presents the results of multivariable linear regressions testing the association of NO_x_ and PM_2.5_ pollution exposure with the secondary outcomes of internalizing, externalizing, and thought disorder symptoms. On average, children exposed to higher levels of continuously measured NO_x_ air pollution displayed greater psychopathology across all psychiatric domains, after adjustment for covariates. Association sizes were weakest for internalizing (fully adjusted b = 1.07; 95% CI, 0.10-2.04; *P* = .03), medium for externalizing (fully adjusted b = 1.42; 95% CI, 0.53-2.31; *P* = .002), and strongest for thought disorder symptoms (fully adjusted b = 1.54; 95% CI, 0.50-2.57; *P* = .004). Use of NO_x_ exposure dichotomized at the top quartile did not change the results (b = 2.62; 95% CI, 0.96-4.27; *P* = .002 for general psychopathology; b = 1.81; 95% CI, 0.16-3.45; *P* = .03 for internalizing symptoms; b = 2.37; 95% CI, 0.81-3.94; *P* = .003 for externalizing symptoms; and b = 3.18; 95% CI, 1.46-4.90: *P* < .001 for though disorder symptoms) (Table 2 and Figure 1).

**Table 2.** Fully Adjusted Association of NO_x_ and PM_2.5_ Air Pollution Exposure in Childhood and Adolescence With the Correlated Factors of Internalizing, Externalizing, and Thought Disorder at 18 Years of Age^a^

Disorder	Air pollution exposure measured continuously and scaled to the interquartile range				Air pollution exposure dichotomized to test extremes (top quartile vs bottom three)			
	NO_x_		PM_2.5_		NO_x_		PM_2.5_	
	b (95% CI)	*P* value	b (95% CI)	*P* value	b (95% CI)	*P* value	b (95% CI)	*P* value
General psychopathology	1.40 (0.41 to 2.38)	.005	0.45 (−0.26 to 1.15)	.22	2.62 (0.96 to 4.27)	.002	2.04 (0.36 to 3.72)	.02
Internalizing	1.07 (0.10 to 2.04)	.03	0.25 (−0.47 to 0.96)	.50	1.81 (0.16 to 3.45)	.03	1.49 (−0.19 to 3.17)	.08
Externalizing	1.42 (0.53 to 2.31)	.002	0.64 (0.02 to 1.26)	.04	2.37 (0.81 to 3.94)	.003	1.54 (−0.01 to 3.09)	.05
Thought disorder	1.54 (0.50 to 2.57)	.004	0.51 (−0.23 to 1.24)	.18	3.18 (1.46 to 4.90)	<.001	2.50 (0.75 to 4.25)	.005

Abbreviations: NO_x_, a regulated gaseous pollutant composed of nitric oxide and nitrogen dioxide; PM_2.5_, a regulated aerosol pollutant with suspended solid and liquid particles smaller than 2.5 μm in diameter.

^a^ The table presents analyses conducted in the full analytic sample of participants with complete air pollution and psychopathology data (analytic sample N = 2039). The b coefficients represent unit change in psychopathology factor scores per interquartile range increment increase in pollutant exposure and moving from the bottom 3 quartiles of air pollutant exposure to the top quartile. General psychopathology was standardized to a mean (SD) of 100 (15). The nonindependence of children within families was accounted for in all models by adjusting the SEs.

For continuously measured PM_2.5_, associations with the secondary outcomes were only significant with externalizing symptoms (fully adjusted b = 0.64; 95% CI, = 0.02-1.26; *P* = .04), after adjustment for covariates. However, use of the dichotomized PM_2.5_ measure revealed that this pollutant was also significantly associated with thought disorder symptoms (fully adjusted b = 2.50; 95% CI, 0.75-4.25; *P* = .005) when pollutant extremes are taken into account (Table 2 and Figure 1).

Next, given the high correlation between NO_x_ and PM_2.5_ (*r* = 0.83, *P* < .001), the primary tests of air pollution–general psychopathology associations were rerun with the inclusion of both pollutants in the fully adjusted model to test for independence of pollutant statistical effects, using the significant dichotomized version of the pollutant measures. In this copollutant model, neither pollutant remained statistically significantly associated with the primary outcome. However, the strength of the association of NO_x_ with general psychopathology was barely attenuated (copollutant-adjusted b = 2.54 vs original b = 2.62, 3% attenuation), whereas the PM_2.5_ association was fully attenuated (copollutant-adjusted b = 0.10 vs original b = 2.04, 95% attenuation). Given this finding, only NO_x_ was carried forward to the second analytic stage.

Finally, sensitivity test results (eTable 3 in the Supplement) found that associations were similar when exposures at 10 and 18 years of age were considered separately and that removing participants who moved before 10 years of age or between 10 and 18 years of age did not change the results (eg, b = 1.40 for the association of NO_x_ and psychopathology in the full analytic sample and b = 1.41 in the subsample of participants who did not move before the age of 10 years).

### Accounting for Correlated Disadvantageous Neighborhood Characteristics

**Figure 2.**
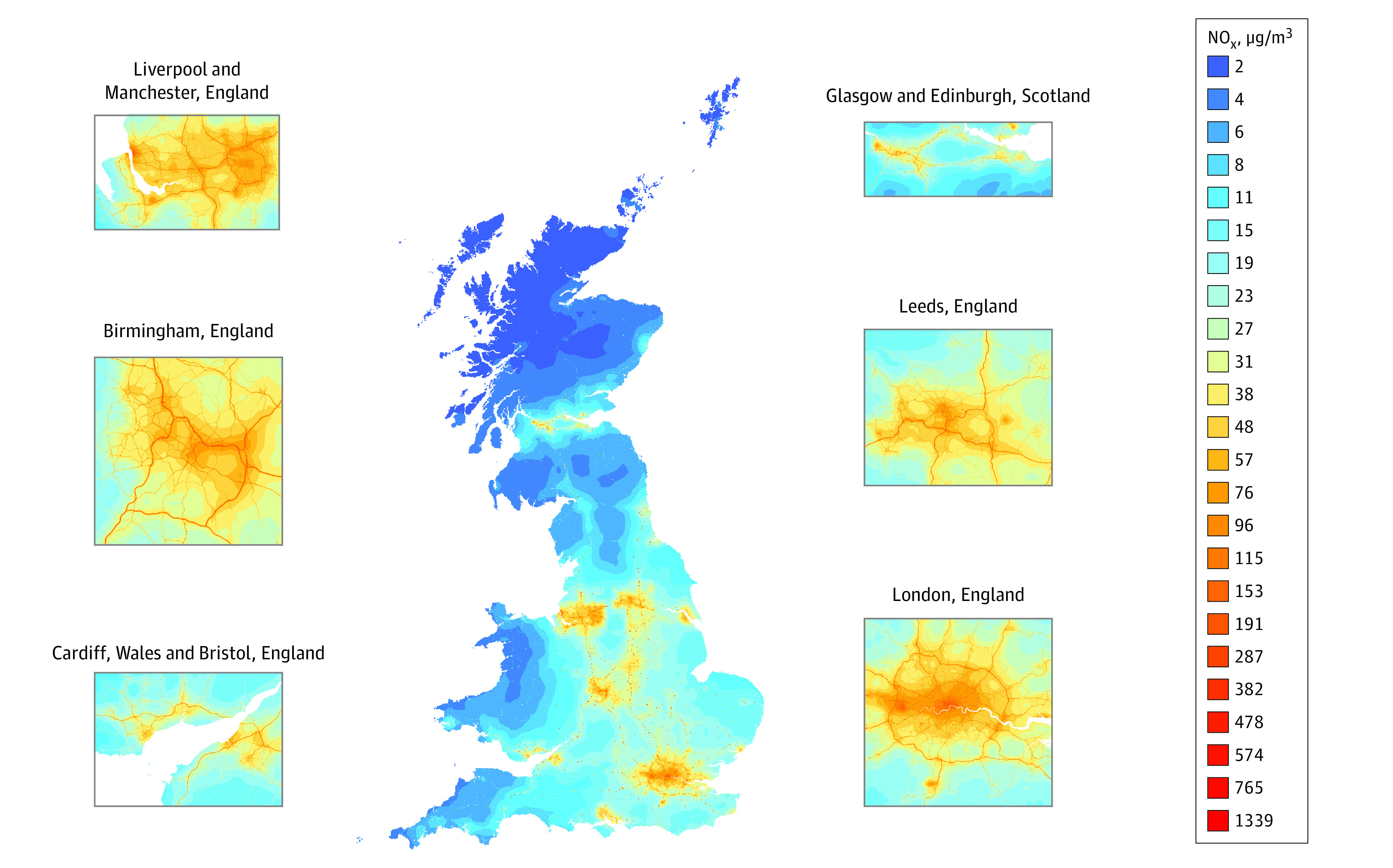
Mean Annual Concentrations of Nitrogen Oxides (NO_x_) Outdoor Air Pollution Across the United Kingdom Annual concentrations are averaged across 2004 and 2012. Inserts depict concentrations in major cities, including London, England.

Figure 2 presents the mean annual NO_x_ concentration across the United Kingdom during the study window, showing higher NO_x_ levels within urban areas and along busy roadways. Annual NO_x_ concentrations across participants’ childhoods were higher in neighborhoods with worse physical, social, and economic conditions (Pearson *r*’s between 0.25 and 0.45 for the association of NO_x_ with neighborhood characteristics) (eTable 4 in the Supplement). This finding raised the possibility that air pollution–psychopathology associations could be driven by the presence of correlated disadvantageous neighborhood characteristics that are also associated with psychopathology.

**Figure 3.**
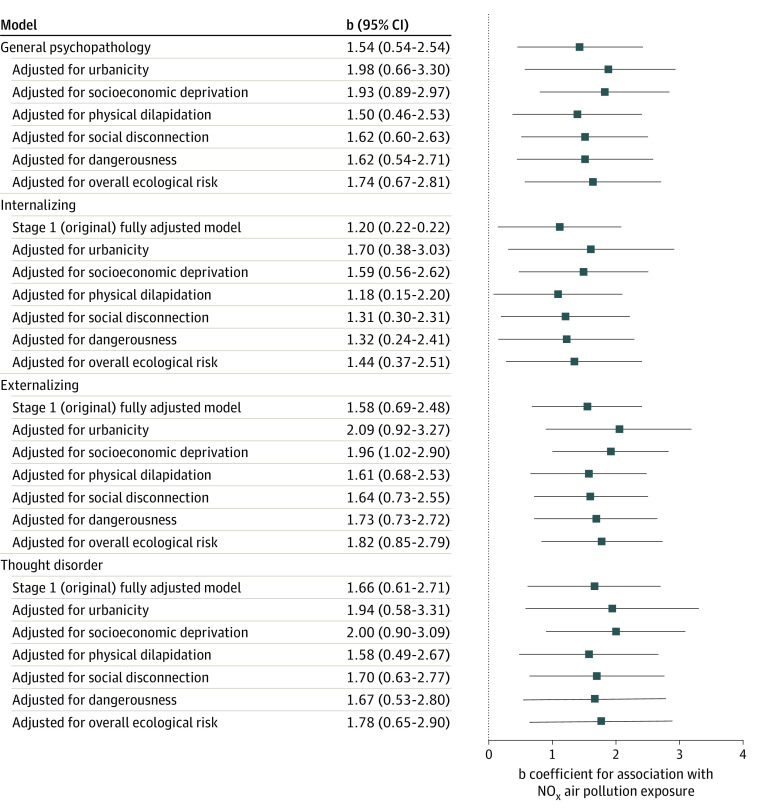
Association of Nitrogen Oxides (NO_x_) Exposure With Psychopathology, Adjusting for Disadvantageous Neighborhood Characteristics Overall ecological risk represents a composite of all disadvantageous neighborhood characteristics (socioeconomic deprivation, physical dilapidation, social disconnection, and dangerousness) measured using geodemographic data from local governments, official crime data from the UK Police, Google street view–based systematic social observation, and surveys of neighborhood residents. The b coefficients represent unit change in psychopathology factor scores at 18 years of age per interquartile range increment increase in NO_x_ exposure in childhood and adolescence. All models were adjusted for sex, family socioeconomic status, family psychiatric history, participant history of emotional and behavioral problems, and tobacco smoking. The nonindependence of children within families was accounted for by adjusting the SEs. Boxes represent the point estimates, and horizontal lines represent the 95% CIs.

Adding each measure of the neighborhood environment to the fully adjusted psychopathology models one at a time and then collectively via the overall Ecological Risk Index did not change the results (Figure 3; eTable 5 in the Supplement). Adjustment for urbanicity also did not change the results, with NO_x_ exposure, continuously measured or dichotomized at the top quartile, remaining significantly associated with all outcomes.

## Discussion

This longitudinal cohort study of air pollution exposure in childhood and adolescence and psychopathology at the transition to adulthood generated 2 main findings. First, youths exposed to higher levels of NO_x_ and PM_2.5_ air pollution had greater levels of general psychopathology at 18 years of age, driven primarily by greater rates of externalizing and thought disorder symptoms. Copollutant models identified NO_x_ as the most robust factor. Second, NO_x_-psychopathology associations were found to be independent of urbanicity; individual and family risks, such as family psychiatric history; and disadvantageous neighborhood characteristics correlated with air pollution, including deprivation, dilapidation, disconnection, and dangerousness.

These results collectively suggest that youths persistently exposed to moderate levels of NO_x_ air pollution may experience greater overall liability to psychiatric illness by young adulthood—a liability independent of other individual, family, and neighborhood influences on mental health. The association of NO_x_ with psychopathology was modest (*r* = 0.08), suggesting that outdoor air pollution is unlikely to be a major etiologic factor in an individual’s psychiatric illness risk across short time spans.^38^ For comparison, the association size is less than half of that seen, in the E-Risk study cohort, for the well-described nonmodifiable risk factor of family history of mental illness (*r* = 0.21). Nevertheless, the association size matches that of other neurotoxicants implicated in psychopathology risk, particularly lead (*r* ≈ 0.08).^39^ Notably, when exposures are widespread, even small elevations in risk can result in significant increases in the burden of disease at the population level.^40^ The WHO estimates that 91% of the global population is exposed to outdoor air pollutants in excess of current guidelines.^41^ Given the ubiquity of this exposure, air pollution could represent a meaningful contributor to the global burden of psychiatric illness, particularly in poor air-quality regions.

What mechanisms could explain associations of early life air pollution exposure with elevations in the p-factor of general psychopathology at the transition to adulthood? First, elevations in the p-factor reported here replicate the general trend of distinct mental illness risk factors leading to nondistinct symptoms. Family history of mental illness and exposure to childhood adversity both robustly elevate risk of mental illness, but with little specificity.^25^ One previously proposed hypothesis to explain these findings is that disruptions to effective CNS development, whether genetic or environmental, result, along a gradient, in less effective control over emotions, reflecting more difficulties in inhibiting negative emotions and cognitive and behavioral responses to emotions.^25^ This ineffective control can arise alongside other markers of impaired CNS development, such as lower cognitive function, which has also been reported, along a gradient, among children exposed to outdoor air pollutants.^42,43^ Overall, robust but nonspecific elevations in psychopathology after air pollution exposure reflect the findings that air pollutants can effect diverse and diffuse CNS developmental impairments, depending on the pollutant mix, duration of exposure, age of exposure, and pathway to the brain (direct or indirect).^33,44^

### Limitations

This study has limitations. First, pollutant-exposure estimates were modeled and not based on personal exposure monitoring, although the model achieves much higher spatial resolution than most studies in the field (20 × 20 m). Second, prenatal and preschool exposures were not measured, although pollutant trends were consistent across the ages of 10 and 18 years, and removal of participants who moved before 10 years of age did not alter results. Third, this study used only measures of NO_x_ and PM_2.5_ and cannot inform about risks related to other criteria pollutants, notably carbon monoxide. Relatedly, our measure of NO_x_ could be a marker of other, unmeasured traffic-related air pollutants,^45^ such as elemental carbon and lead. Because lead was not banned from gasoline in the UK until 2000 (at approximately 6 years of age for E-Risk study children), participants with higher NO_x_ exposure also likely had higher early life lead exposure.^46^ Fourth, we were unable to rule out traffic-related noise, which has been associated with poor psychiatric outcomes.^47,48^ Fifth, the extent to which these findings would generalize to contexts with extremely high pollutant concentrations (eg, China, India, and Nepal) is unknown. Sixth, this study was observational and cannot establish causation, although it was able to use high-quality measurements of covariates to address important alternate explanations at the individual, family, and neighborhood levels.

## Conclusions

In this longitudinal cohort study, youths exposed to higher levels of NO_x_ air pollution in childhood and adolescence experienced greater psychopathology at the transition to adulthood. These findings suggest that air pollution may be a nonspecific risk factor for the development of psychopathology.

## Notes

Supplement. **eFigure 1.** Distribution of E-Risk Study Families’ Addresses Across England and Wales **eFigure 2.** The E-Risk Study Families’ Addresses Are a Near-Perfect Match to the Deciles of the UK Government’s Index of Multiple Deprivation **eFigure 3.** The Structure of Psychopathology at Age 18 Years in the E-Risk Cohort **eTable 1.** Family and Individual-Level Sociodemographic Characteristics of the E-Risk Participants at Age 18 Years (Panel A) and Participant Neighborhood Characteristics (Panel B) **eTable 2.** Performance Statistics for the Air Pollution Exposure Estimates **eTable 3.** Association of NO_x_ and PM_2.5_ Air Pollution Exposure at Ages 10 and 18 With General Psychopathology and the Correlated Factors of Internalizing, Externalizing, and Thought Disorder at Age 18 Years **eTable 4.** Correlation of NO_x_ and PM_2.5_ Air Pollution With Neighborhood Urbanicity and Disadvantageous Neighborhood Characteristics **eTable 5.** Association of NO_x_ Air Pollution Exposure With General Psychopathology and Internalizing, Externalizing, and Thought Disorder Accounting for Correlated Disadvantageous Neighborhood Characteristics **eAppendix 1.** Additional Details on the E-Risk Study Sample **eAppendix 2.** Additional Details on the Measurement of Air Pollution Exposure **eAppendix 3.** Additional Details of the Measures of Psychopathology **eAppendix 4.** Additional Details on the Study Covariates **eAppendix 5.** Additional Details on the Disadvantageous Neighborhood Characteristics and Measurement of Urbanicity **eAppendix 6.** Comparison of Participants With and Without Complete Air Pollution and Psychopathology Data **eReferences**
