# Supplementary material for: Association of Air Pollution Exposure in Childhood and Adolescence With Psychopathology at the Transition to Adulthood
Source: JAMA Netw Open. 2021 Apr 28;4(4):e217508. doi: 10.1001/jamanetworkopen.2021.7508 (PMC8082321; doi:10.1001/jamanetworkopen.2021.7508)
Supplement: Supplement. — eFigure 1. Distribution of E-Risk Study Families’ Addresses Across England and Wales eFigure 2. The E-Risk Study Families’ Addresses Are a Near-Perfect Match to the Deciles of the UK Government’s Index of Multiple Deprivation eFigure 3. The Structure of Psychopathology at Age 18 Years in the E-Risk Cohort eTable 1. Family and Individual-Level Sociodemographic Characteristics of the E-Risk Participants at Age 18 Years (Panel A) and Participant Neighborhood Characteristics (Panel B) eTable 2. Performance Statistics for the Air Pollution Exposure Estimates eTable 3. Association of NOx and PM2.5 Air Pollution Exposure at Ages 10 and 18 With General Psychopathology and the Correlated Factors of Internalizing, Externalizing, and Thought Disorder at Age 18 Years eTable 4. Correlation of NOx and PM2.5 Air Pollution With Neighborhood Urbanicity and Disadvantageous Neighborhood Characteristics eTable 5. Association of NOx Air Pollution Exposure With General Psychopathology and Internalizing, Externalizing, and Thought Disorder Accounting for Correlated Disadvantageous Neighborhood Characteristics eAppendix 1. Additional Details on the E-Risk Study Sample eAppendix 2. Additional Details on the Measurement of Air Pollution Exposure eAppendix 3. Additional Details of the Measures of Psychopathology eAppendix 4. Additional Details on the Study Covariates eAppendix 5. Additional Details on the Disadvantageous Neighborhood Characteristics and Measurement of Urbanicity eAppendix 6. Comparison of Participants With and Without Complete Air Pollution and Psychopathology Data eReferences [file jamanetwopen-e217508-s001.pdf]

Supplementary Online Content

Reuben A, Arseneault L, Beddows A, et al. Association of air pollution exposure in childhood and adolescence with psychopathology at the transition to adulthood. *JAMA Netw Open*. 2021;4(4):e217508. doi:10.1001/jamanetworkopen.2021.7508

eFigure 1. Distribution of E-Risk Study Families' Addresses Across England and Wales
eFigure 2. The E-Risk Study Families' Addresses Are a Near-Perfect Match to the Deciles of the UK Government's Index of Multiple Deprivation
eFigure 3. The Structure of Psychopathology at Age 18 Years in the E-Risk Cohort
eTable 1. Family and Individual-Level Sociodemographic Characteristics of the E-Risk Participants at Age 18 Years (Panel A) and Participant Neighborhood Characteristics (Panel B)
eTable 2. Performance Statistics for the Air Pollution Exposure Estimates
eTable 3. Association of NO_x and $PM_{2.5}$ Air Pollution Exposure at Ages 10 and 18 With General Psychopathology and the Correlated Factors of Internalizing, Externalizing, and Thought Disorder at Age 18 Years
eTable 4. Correlation of NO_x and $PM_{2.5}$ Air Pollution With Neighborhood Urbanicity and Disadvantageous Neighborhood Characteristics
eTable 5. Association of NO_x Air Pollution Exposure With General Psychopathology and Internalizing, Externalizing, and Thought Disorder Accounting for Correlated Disadvantageous Neighborhood Characteristics
eAppendix 1. Additional Details on the E-Risk Study Sample
eAppendix 2. Additional Details on the Measurement of Air Pollution Exposure
eAppendix 3. Additional Details of the Measures of Psychopathology
eAppendix 4. Additional Details on the Study Covariates
eAppendix 5. Additional Details on the Disadvantageous Neighborhood Characteristics and Measurement of Urbanicity
eAppendix 6. Comparison of Participants With and Without Complete Air Pollution and Psychopathology Data
eReferences

This supplementary material has been provided by the authors to give readers additional information about their work.

© 2021 Reuben A et al. *JAMA Network Open.*

eFigure 1. Distribution of E-Risk Study families' addresses across England and Wales.

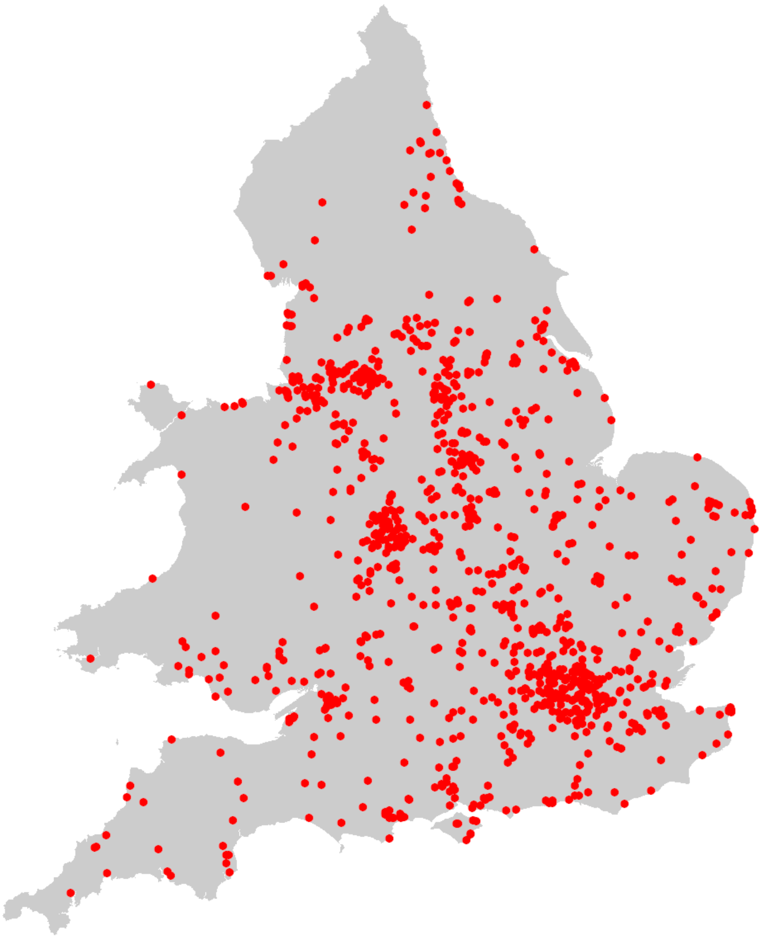

Figure displays E-Risk Study participants' home addresses at the age 12 assessment.[1]

© 2021 Reuben A et al. *JAMA Network Open.*

eFigure 2. The E-Risk Study families' addresses are a near-perfect match to the deciles of the UK Government's Index of Multiple Deprivation.

This histogram shows E-Risk families' addresses are a near-perfect match to the deciles of the UK's 2015 Lower-layer Super Output Area (LSOA) Index of Multiple Deprivation (IMD) which averages 1,500 residents; approximately 10% (dotted red line) of the E-Risk cohort fills each of the IMD's 10% bands, indicating that the E-Risk cohort accurately represents the distribution of deprivation in the UK.

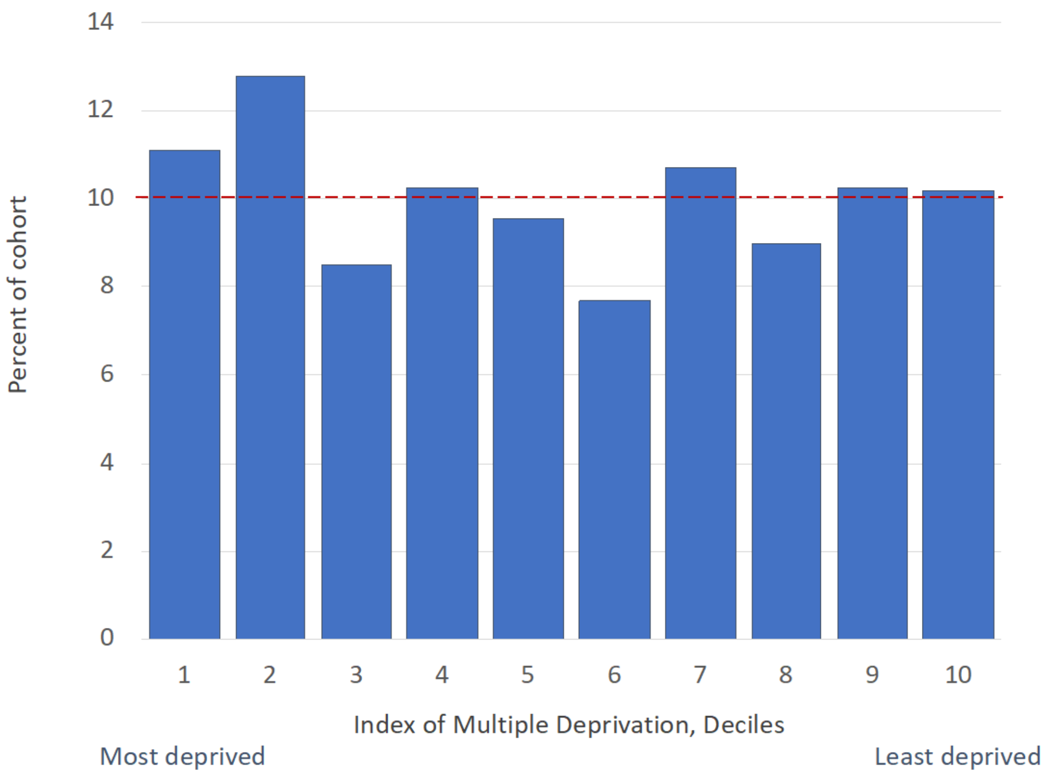

Note. The UK Ministry of Housing, Communities & Local Government Index of Multiple Deprivation is an official measure of relative deprivation for every LSOA small area (approximately 1,500 residents or 650 households each) in England.

© 2021 Reuben A et al. *JAMA Network Open.*

eFigure 3. The structure of psychopathology at age 18 years in the E-Risk Cohort.

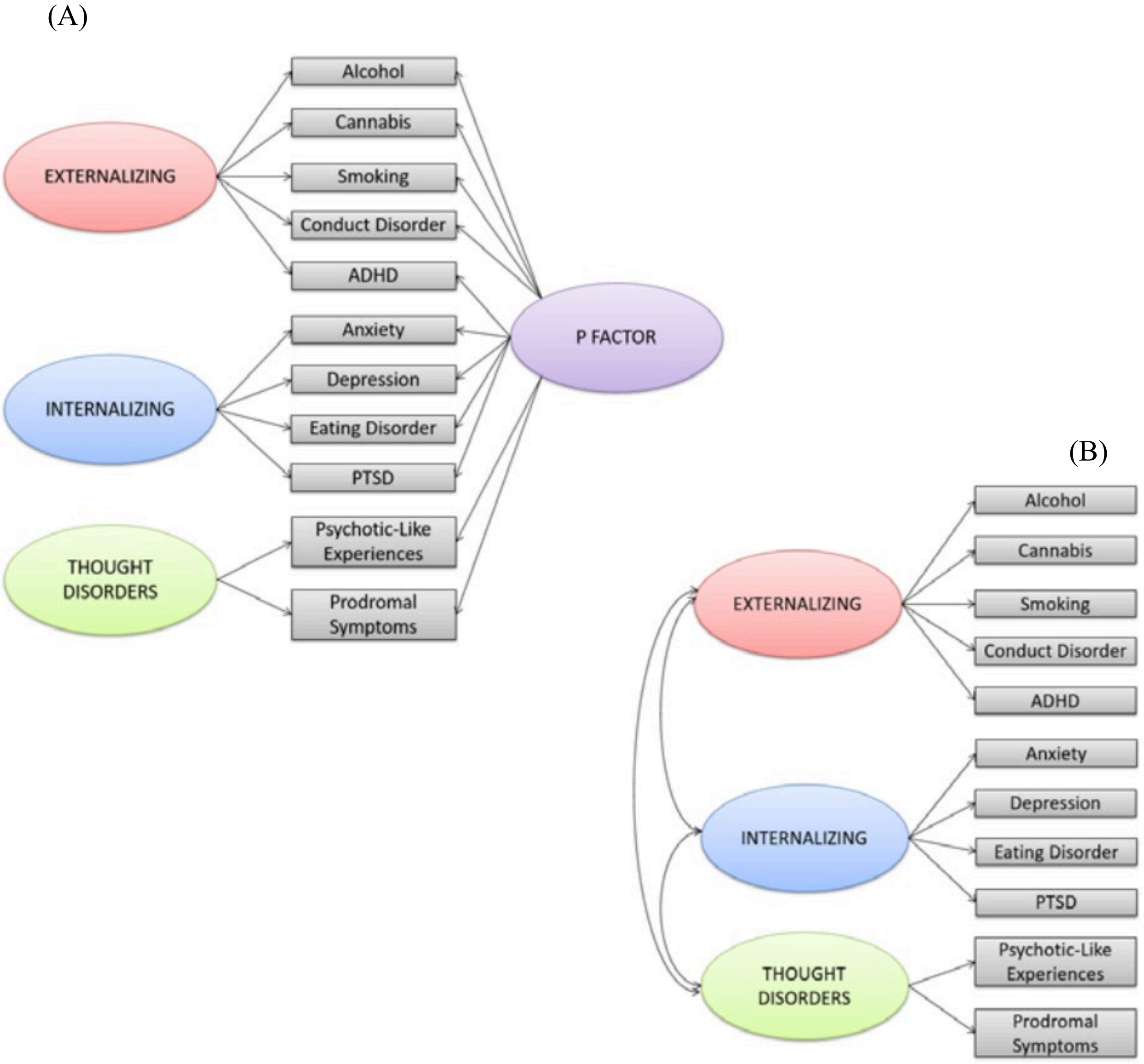

Note. (A) Bi-factor model, (B) Correlated-factors model. Colored ovals represent latent (unobserved) continuous symptom trait factors; grey boxes represent age-18 observed scores on symptom scales corresponding to each disorder. ADHD = attention-deficit/hyperactivity disorder, PTSD = post-traumatic stress disorder. P-Factor represents the factor of General Psychopathology. Figure reproduced from Schaefer et al.[5]

© 2021 Reuben A et al. *JAMA Network Open.*

eTable 1. Family and individual-level sociodemographic characteristics of the E-Risk participants at age 18 years (Panel A) and participant neighborhood characteristics (Panel B).

(A)	**Full analytic sample**		**Per quartile of NO_x in childhood and adolescence (range in µg/m^3)**			
			1st Quartile (2.45-18.90)	2nd Quartile (18.9-26.73)	3rd Quartile (26.74-38.74)	4th Quartile (38.75- 113.07)
Total, No. (%)	2039 (100)		533 (26.1)	515 (25.3)	507 (24.9)	484 (23.7)
Sex, No. (%)						
Female	1071 (52.5)		280 (52.5)	274 (53.2)	282 (55.6)	268 (55.4)
Male	968 (47.5)		253 (47.5)	241 (46.8)	225 (44.4)	216 (44.6)
Family SES, No. (%)						
Low	691 (33.9)		168 (31.5)	165 (32.0)	156 (30.1)	202 (41.7)
Middle	676 (33.2)		159 (29.8)	184 (35.7)	174 (34.3)	159 (32.9)
High	672 (33.0)		203 (38.7)	166 (32.2)	177 (34.9)	123 (25.4)
Family psychiatric history, scaled 0-1.0, mean (SD)	0.37 (0.27)		0.39 (0.26)	0.35 (0.27)	0.37 (0.26)	0.38 (0.28)
Participant history of emotional and behavioral problems in early childhood, z-score units, mean (SD)	0.00 (0.80)		-0.09 (0.68)	0.01 (0.83)	-0.02 (0.81)	0.11 (0.86)
Participant tobacco smoking, pack years, mean (SD)	0.53 (1.23)		0.55 (1.36)	0.51 (1.10)	0.54 (1.32)	0.50 (1.11)

© 2021 Reuben A et al. *JAMA Network Open.*

(B)	Full analytic sample		**Per quartile of NO_x in childhood and adolescence (range in µg/m^3)**			
			1st Quartile (2.45 - 18.90)	2nd Quartile (18.91 - 26.73)	3rd Quartile (26.74 - 38.74)	4th Quartile (38.75 - 113.07)
Total, No. (%)	2039 (100)		533 (26.1)	515 (25.3)	507 (24.9)	484 (23.7)
Urbanicity, No. (%)						
Rural	400 (20.2)		221 (44.5)	104 (20.7)	59 (11.9)	16 (3.3)
Intermediate	942 (47.7)		275 (55.3)	369 (73.4)	225 (45.5)	73 (15.2)
Urban	635 (32.1)		1 (0.2)	30 (6.0)	211 (42.6)	393 (81.5)
Neighborhood Characteristics						
Deprived, z-score units, mean (SD)	0.00 (1.01)		-0.36 (0.88)	-0.18 (0.86)	-0.02 (0.98)	0.62 (1.04)
Dilapidated, z-score units, mean (SD)	0.00 (1.01)		-0.27 (0.90)	-.19 (0.89)	-0.03 (1.02)	0.51 (1.02)
Disconnected, z-score units, mean (SD)	0.00 (1.01)		-0.27 (0.92)	-.10 (0.95)	-0.02 (0.97)	0.41 (1.06)
Dangerous, z-score units, mean (SD)	-0.01 (1.01)		-0.52 (0.98)	-0.15 (0.90)	0.04 (0.86)	0.65 (0.89)
Overall Ecological Risk, mean(SD)	200.00 (33.49)		185.76 (28.58)	193.76 (29.90)	199.64 (31.81)	222.25 (32.53)

Note. NO_x = a regulated gaseous pollutant composed of nitric oxide and nitrogen dioxide. SD = standard deviation. SES = socioeconomic status. Overall Ecological Risk represents a composite of all disadvantageous neighborhood characteristics (socioeconomic deprivation, physical dilapidation, social disconnection, and dangerousness) measured using geodemographic data from local governments, official crime data from the UK Police, Google Street View-based Systematic Social Observation, and surveys of neighborhood residents. Neighborhood characteristics are presented in z-score units but were scaled to mean(SD)=50(10) for summation into the Ecological Risk index. Urbanicity data was present for 1,977 (97.0%) participants in the full analytic sample; neighborhood characteristic data was present for 1,994 (97.8%) to 2,029 (99.5%) participants in the full analytic sample.

© 2021 Reuben A et al. *JAMA Network Open.*

eTable 2. Performance statistics for the air pollution exposure estimates.

Pollutant	Year	Assessment age	Number of data points	Observed mean (µg/m^3)	Modeled mean (µg/m^3)	FAC2	MB (µg/m^3)	NMB	RMSE (µg/m^3)	r
NO_x	2004	10	189	97.72	86.74	0.92	-10.90	-0.11	42.76	0.82
	2012	18	109	81.46	74.99	0.94	-6.47	-0.08	30.52	0.89
$PM_{2.5}$	2004	10	8	17.48	16.78	1	-0.70	-0.04	3.54	0.66
	2012	18	86	13.03	12.62	1	-0.41	-0.03	2.80	0.66

Note. FAC2 = fraction of predictions within a factor of two; MB = mean bias; NMB = normalised mean bias; RMSE = root mean squared error; r = correlation coefficient between observed and modeled.

© 2021 Reuben A et al. *JAMA Network Open.*

eTable 3. Association of NO_x and $PM_{2.5}$ air pollution exposure at ages 10 and 18 with General Psychopathology and the correlated factors of Internalizing, Externalizing, and Thought Disorder at age 18 years.

	Age 10 exposure						Age 18 exposure				
	**NO_x**			**$PM_{2.5}$**			**NO_x**			**$PM_{2.5}$**	
	**b (95% CI)**	***P*-value**		**b (95% CI)**	***P*-value**		**b (95% CI)**	***P*-value**		**b (95% CI)**	***P*-value**
General Psychopathology	2.54 (0.86, 4.22)	.003		1.78 (0.10, 3.47)	.038		2.71 (1.09, 4.33)	.001		1.67 (0.04, 3.30)	.044
Internalizing	2.06 (0.38, 3.73)	.016		1.57 (-0.11, 3.25)	.067		1.78 (0.16, 3.40)	.031		1.06 (-0.56, 2.68)	.201
Externalizing	1.70 (0.09, 3.31)	.038		1.22 (-0.36, 2.81)	.130		2.63 (1.12, 4.14)	.001		1.50 (-0.03, 3.03)	.054
Thought Disorder	3.02 (1.28, 4.75)	.001		1.97 (0.24, 3.70)	.026		3.34 (1.65, 5.03)	<.001		2.13 (0.44, 3.82)	.013

Note. CI = confidence interval. NO_x = a regulated gaseous pollutant composed of nitric oxide and nitrogen dioxide. $PM_{2.5}$ = a regulated aerosol pollutant with suspended solid and liquid particles smaller than 2.5 microns in diameter. All associations report the "fully adjusted" model adjusting for sex, family socioeconomic status, family psychiatric history, participant history of emotional and behavioral problems, and tobacco smoking. Beta coefficients represent unit change in psychopathology factor scores when moving from the bottom three quartiles of air pollutant exposure to the top quartile. Psychopathology factors were standardized to mean(SD)=100(15). The non-independence of children within families was accounted for in all models by adjusting the standard errors.

© 2021 Reuben A et al. *JAMA Network Open.*

eTable 4. Correlation of NO_x and $PM_{2.5}$ air pollution with neighborhood urbanicity and disadvantageous neighborhood characteristics.

	**NO_x**	**$PM_{2.5}$**
	Spearman's ρ	
Urbanicity	.67	.50
	Pearson's r	
Socioeconomic deprivation	.36	.21
Physical dilapidation	.30	.18
Social disconnection	.25	.18
Dangerousness	.45	.32
Overall Ecological Risk	.41	.27

Note. All correlations significant at $P<.001$. NO_x = a regulated gaseous pollutant composed of nitric oxide and nitrogen dioxide. $PM_{2.5}$ = a regulated aerosol pollutant with suspended solid and liquid particles smaller than 2.5 microns in diameter. Overall Ecological Risk represents a composite of all disadvantageous neighborhood characteristics (socioeconomic deprivation, physical dilapidation, social disconnection, and dangerousness) measured using geodemographic data from local governments, official crime data from the UK Police, Google Street View-based Systematic Social Observation, and surveys of neighborhood residents.

© 2021 Reuben A et al. *JAMA Network Open.*

eTable 5. Association of NO_x air pollution exposure with General Psychopathology and Internalizing, Externalizing, and Thought Disorder accounting for correlated disadvantageous neighborhood characteristics.

Models	General Psychopathology			Internalizing			Externalizing			Thought Disorder	
	**b (95% CI)**	***P*-value**		**b (95% CI)**	***P*-value**		**b (95% CI)**	***P*-value**		**b (95% CI)**	***P*-value**
Stage 1 (original) fully adusted model	1.54 (0.54, 2.54)	.003		1.20 (0.22, 2.19)	.017		1.58 (0.69, 2.48)	.001		1.66 (0.61, 2.71)	.002
Additionally adjusted for urbanicity	1.98 (0.66, 3.30)	.003		1.70 (0.38, 3.03)	.012		2.09 (0.92, 3.27)	<.001		1.94 (0.58, 3.31)	.005
Additionally adjusted for deprivation	1.93 (0.89, 2.97)	<.001		1.59 (0.56, 2.62)	.002		1.96 (1.02, 2.90)	<.001		2.00 (0.90, 3.09)	<.001
Additionally adjusted for dilapidation	1.50 (0.46, 2.53)	.005		1.18 (0.15, 2.20)	.025		1.61 (0.68, 2.53)	.001		1.58 (0.49, 2.67)	.005
Additionally adjusted for disconnection	1.62 (0.60, 2.63)	.002		1.31 (0.30, 2.31)	.011		1.64 (0.73, 2.55)	<.001		1.70 (0.63, 2.77)	.002
Additionally adjusted for dangerousness	1.62 (0.54, 2.71)	.003		1.32 (0.24, 2.41)	.017		1.73 (0.73, 2.72)	.001		1.67 (0.53, 2.80)	.004
Additionally adjusted for overall Ecological Risk	1.74 (0.67, 2.81)	.001		1.44 (0.37, 2.51)	.008		1.82 (0.85, 2.79)	<.001		1.78 (0.65, 2.90)	.002

Note. CI = confidence interval. NO_x = a regulated gaseous pollutant composed of nitric oxide and nitrogen dioxide. All associations adjusted for sex, family socioeconomic status, family psychiatric history, participant history of emotional and behavioral problems, and tobacco smoking. Beta coefficients represent unit change in psychopathology factor scores per interquartile range increment increase in NO_x. Psychopathology factors were standardized to mean(SD)=100(15). The non-independence of children within families was accounted for in all models by adjusting the standard errors. Table presents analyses conducted under a subset of participants with full air pollution, psychopathology, and neighborhood data (N=1986, 97.4% of full-study analytic sample).

© 2021 Reuben A et al. *JAMA Network Open.*

eAppendix 1. Additional details on the E-Risk Study sample.

Participants are members of the Environmental-Risk (E-Risk) Longitudinal Twin Study, a nationally representative sample of children born in 1994 and 1995 in England and Wales (N=2,232). Briefly, the E-Risk sample was constructed in 1999–2000, when 1116 families with same-sex 5-year-old twins (93% of those eligible) participated in home-visit assessments. The full sample comprised 56% monozygotic (MZ) and 44% dizygotic (DZ) twin pairs; sex was evenly distributed within zygosity (49% male). Families were recruited to represent the UK population of families with newborns in the 1990s, based on residential location throughout England and Wales and mothers' age (teenaged mothers with twins were over-selected to replace high-risk families who were selectively lost to the register through non-response. Older mothers having twins via assisted reproduction were under-selected to avoid an excess of well-educated older mothers). The cohort's neighborhoods represent the full range of socioeconomic conditions in Great Britain, as reflected in the families' distribution on a neighborhood-level socioeconomic index (ACORN [A Classification of Residential Neighborhoods], developed by CACI Inc. for commercial use).[1] E-Risk families' ACORN distribution closely matches that of households nation-wide: 25.6% of E-Risk families live in "wealthy achiever" neighborhoods compared to 25.3% nationwide; 5.3% vs. 11.6% live in "urban prosperity" neighborhoods; 29.6% vs. 26.9% live in "comfortably off" neighborhoods; 13.4% vs. 13.9% live in "moderate means" neighborhoods; and 26.1% vs. 20.7% live in "hard-pressed" neighborhoods. E-Risk underrepresents "urban prosperity" neighborhoods because such households are likely to be childless. **Figure S1** shows E-Risk families' addresses are a near-perfect match to the deciles of the UK

© 2021 Reuben A et al. *JAMA Network Open.*

government's 2015 Lower-layer Super Output Area (LSOA) Index of Multiple Deprivation, which ranks British neighborhoods in terms of relative deprivation at an area level of approximately 1,500 residents; approximately 10% of the E-Risk cohort fills each of the Index's 10% bands, indicating that the E-Risk cohort accurately represents the distribution of deprivation in the UK.

Follow-up home visits were conducted when the participants were aged 7 (98% participation), 10 (96%), 12 (96%), and, most recently, 18 (93%) years. Home visits at ages 5, 7, 10, and 12 years included assessments with participants as well as their mother (or primary caretaker); the home visit at age 18 included structured interviews only with the participants. Each twin participant was assessed by a different interviewer. The average age of the twins at the time of the age-18 assessment was 18.4 years (SD = 0.36); all structured interviews were conducted after the 18th birthday. There were no differences between those who did and did not take part at age 18 in terms of age-5 socioeconomic status (SES) (χ^2=0.86; P=0.65), age-5 IQ scores (t=0.98; P=0.33), or age-5 emotional or behavioral problems (t=0.40; P=0.69 and t=0.41; P=0.68, respectively).

The Joint South London and Maudsley and the Institute of Psychiatry Research Ethics Committee approved each phase of the study. Parents gave informed consent and twins gave assent between 5–12 years and then informed consent at age 18. Further details about the sample are reported elsewhere.[2]

© 2021 Reuben A et al. *JAMA Network Open.*

eAppendix 2. Additional details on the measurement of air pollution exposure.

Ground-based evaluation of the ambient air pollution measures

Air pollution exposure was modeled at the local-scale using the US-Environmental Protection Agency's regional chemical-transport model, the Community Multiscale Air Quality Modeling (CMAQ),[3] coupled with the ADMS street-scale dispersion model, referred to as CMAQ-urban.[4] CMAQ-urban utilized emissions data from the UK National Atmospheric Emissions Inventory and the Imperial College's newest generation of UK road-traffic emissions inventory.

The modelled annual mean concentrations of NO_x and $PM_{2.5}$ were assessed via ground-based evaluation prior to their use in this study. Modeled estimates were compared against ground-based measurements obtained from the Automatic Urban and Rural Network (AURN) and London Air Quality Network (LAQN) which include rural (16), urban background (81), roadside (49), kerbside (8), and industrial (4) sites. Performance statistics (**Table S2**) showed good percentages of predictions within a factor of two of the measurements (FAC2 × 100), i.e., 96% for NO_x and 100% for $PM_{2.5}$. Overall the model slightly underestimates NO_x (10.90μg/m^3 or 11% in 2004 [age-10] and 6.47μg/m^3 or 8% in 2012 [age 18]) and $PM_{2.5}$ (0.70μg/m^3 or 4% in 2004 and 0.41μg/m^3 or 3% in 2012)**.** RMSE and r reveal that the spatial variations of the predicted NO_x (RMSE=30.52 to 42.76μg/m^3, r=0.82 to 0.89) are reasonably accurate although slightly less so for $PM_{2.5}$ (RMSE=2.80 to 3.54μg/m^3, r=0.66 at both time points). Further investigation has revealed that the prediction bias is largest at industrial and kerbside locations where emissions estimates are highly uncertain. Tackling uncertainties in emissions would further enhance the model performance.

© 2021 Reuben A et al. *JAMA Network Open.*

eAppendix 3. Additional details on the measures of psychopathology.

Assessment of symptoms of mental disorder

At age 18, participants were assessed in private interviews about past-year symptoms of mental disorders.[5] Five externalizing-spectrum disorder symptoms were assessed: Diagnostic and Statistical Manual of Mental Disorders 4th edition (DSM–IV)[6] symptoms of alcohol dependence and cannabis dependence assessed via the Diagnostic Interview Schedule (DIS);[7] conduct disorder assessed by inquiring about DSM–IV symptoms; symptoms of tobacco dependence assessed via the Fagerstrom Test for Nicotine Dependence;[8] and attention-deficit/ hyperactivity disorder (ADHD) assessed by inquiring about DSM 5th edition (DSM–5)[9] symptoms.[10] Four internalizing-spectrum disorder symptoms were assessed: DSM–IV symptoms of depression, generalized anxiety disorder, and posttraumatic stress disorder (PTSD) assessed via the DIS,[7] and symptoms of eating disorder assessed via the SCOFF.[11] Thought disorder symptoms were assessed in two ways: first, participants were asked 7 items about delusions and hallucinations (psychotic-like experiences: e.g., "Have other people ever read your thoughts?"; "Have you ever thought you were being followed or spied on?"; "Have you ever heard voices that other people cannot hear?").[12] Second, participants were asked 6 items about unusual thoughts and feelings (prodromal symptoms: e.g., "My thinking is unusual or frightening"; "People or places I know seem different"), drawing on item pools since formalized in prodromal psychosis instruments, including the PRIME-screen and SIPS.[13]

The structure of psychopathology

Using confirmatory factor analysis, two standard models[14,15] that are frequently used to examine hierarchically structured constructs were estimated: a correlated-factors

© 2021 Reuben A et al. *JAMA Network Open.*

model with three factors (representing Internalizing, Externalizing, and Thought Disorder symptoms) and a bi-factor model specifying a General Psychopathology factor (**Figure S3**) in addition to the three specific factors. Decisions about symptom-factor loadings were guided by the Hierarchical Taxonomy of Psychopathology consortium (https://medicine.stonybrookmedicine.edu/HITOP/AboutHiTOP).[16] Symptoms corresponding to disorders of distress (depression, generalized anxiety disorder, and PTSD) and eating pathology loaded on the Internalizing factor; symptoms corresponding to disorders of substance use (alcohol, cannabis, tobacco) and oppositional behavior (conduct disorder) and ADHD loaded on the Externalizing factor; and symptoms corresponding to disorders associated with psychosis loaded on the Thought Disorder factor. Confirmatory factor analyses were run as two-level clustered models to account for the nesting of twins within families, with analyses performed in MPlus v7.4[17] using the robust maximum likelihood estimator (MLR) to provide standard errors that are robust to non-normality and non-independence of observations.

Both models fit the data well as assessed by the Akaike Information Criterion (AIC), Bayesian Information Criterion (BIC) and the Sample Adjusted BIC, although the bi-factor model demonstrated marginally superior fit.

For the correlated-factors model, AIC=42987.116, BIC=43488.486, Sample Adjusted BIC=43205.726. Loadings on each of the three factors were all positive, generally high (all p's < .001) and averaged 0.680 (Externalizing: average loading=0.638; Internalizing: average loading=0.654; Thought Disorder: average loading=0.836). Correlations between the three factors were all positive and ranged from 0.552 between Externalizing and Thought Disorder to 0.756 between Internalizing and

© 2021 Reuben A et al. *JAMA Network Open.*

Thought Disorder. Thus, this model confirmed that three correlated factors (i.e., Internalizing, Externalizing, and Thought Disorder) explained the structure of the 11 symptom scales examined in the E-Risk twins at age 18.

For the bi-factor model, AIC=42897.350, BIC=43443.787, Sample Adjusted BIC=43135.609. Loadings on the General Psychopathology factor (“p”) were all positive, generally high (all p’s < .001) and averaged 0.519; the highest standardized loadings were for psychotic symptoms (0.759 and 0.592), major depressive episode (0.718), eating disorders (0.574), and generalized anxiety disorder (0.567). Similarly, the loadings for the three style factors were all positive and averaged 0.507 for Externalizing, 0.270 for Internalizing, and 0.496 for Thought Disorder. Thus, this model confirmed that a bi-factor structure (i.e., with a General Psychopathology factor and three specific Internalizing, Externalizing, and Thought Disorder factors) explained the structure of the 11 symptom scales examined in the E-Risk twins at age 18.

© 2021 Reuben A et al. *JAMA Network Open.*

eAppendix 4. Additional details on the study covariates.

Covariates in the fully adjusted model included biological sex at birth and:

Family socioeconomic status, measured via a composite of parental income, education, and occupation measured when participants were age 5. The three SES indicators were highly correlated (r's ranged from .57 to .67, *P*-value's <.05) and loaded significantly onto one latent factor (factor loadings=0.80, 0.70, and 0.83 for income, education, and occupation, respectively). The latent variable was categorized into tertiles (i.e., low–, medium–, and high–SES).[18]

Family psychiatric history, assessed at the age-12 assessment from reports by biological mothers conducted as part of a family history interview.[19] Family history of psychiatric disorder was defined as a report of treatment or hospitalization for a psychiatric disorder or substance-use problem, or attempted or completed suicide for any of the child's biological mother, father, grandparents, or aunts and uncles. This was converted to the proportion of family members with a history of any psychiatric disorder (coded 0–1.0; cohort mean(SD)=0.37(0.27)).

Participant history of emotional and behavioral problems in early childhood (age 5). Emotional and behavioral problems at age 5 were assessed using the Child Behavior Checklist in interviews with mothers and the Teacher Report Form by mail for teachers.[20,21] The emotional (internalizing) problems scale is the sum of items in the withdrawn and anxious/depressed subscales, and the behavioral (externalizing) problems scale is the sum of items from the aggressive and delinquent behavior subscales. We summed and standardized mothers' and teachers' reports of each of these measures to create cross-informant scales representing total emotional and behavioral problems.

© 2021 Reuben A et al. *JAMA Network Open.*

Tobacco smoking, measured as total pack years smoked, by age 18, reported by participants at the age-18 assessment.

© 2021 Reuben A et al. *JAMA Network Open.*

eAppendix 5. Additional details on the disadvantageous neighborhood characteristics and measurement of urbanicity.

Disadvantageous neighborhood characteristics were measured through ecological risk assessment conducted by combining information from four independent sources of data: 1) geodemographic data from local governments; 2) official crime data from the UK Police; 3) Google Street View-based Systematic Social Observation (SSO);[22] and, 4) surveys of neighborhood residents having the same postcode as each Study family, conducted by the E-Risk Study team. We used these data sources to measure four neighborhood characteristics across childhood (from ages 5 to 17 years): deprivation, dilapidation, disconnection, and dangerousness. For each characteristic, a measure of ecological risk was constructed. First, variables with skewed distributions were log-transformed. Second, values were standardized to mean(SD)=50(10). Third, mean scores were taken across measurement method within each domain. The resulting scales of deprivation, dilapidation, disconnection, and dangerousness were approximately normally distributed. Neighborhoods' ecological risk levels on these four measures were correlated (r=0.5-0.7). An overall composite Ecological-Risk Index was created by summing values across the four measures. Urbanicity was also separately categorized.

Neighborhood characteristics measured

Deprivation was measured with the UK Ministry of Housing, Communities & Local Government Index of Multiple Deprivation, an official measure of relative deprivation for every Lower-layer Super Output Area (LSOA) small area (approximately 1,500 residents or 650 households each) in England.

© 2021 Reuben A et al. *JAMA Network Open.*

Dilapidation was measured from resident ratings of problems in their neighborhood (e.g. litter, vandalized public spaces, vacant storefronts) and independent raters' assessments of these same problems based on the "virtual walk-through" using Google Street View.

Disconnection was measured from resident ratings assessing neighborhood collective efficacy and social connectedness. *Neighborhood collective efficacy* was assessed via the resident survey using a previously validated 10-item measure of social control and social cohesion.[23] Residents were asked about the likelihood that their neighbors could be counted on to intervene in various ways if, for example: "children were skipping school and hanging out on a street corner", "children were spray-painting graffiti on a local building." They were also asked how strongly they agreed that, for example: "people around here are willing to help their neighbors", "this is a close-knit neighborhood" (item responses: 0-4). *Social connectedness* was assessed based on indicators of *intergenerational closeness* ("If any of your neighbors' children did anything that upset you would you feel that you could speak to their parents about it?"), *reciprocated exchange* (e.g., "Would you be happy to leave your keys with a neighbor if you went away on holiday?"), and *friendship ties* (e.g., "Do you have any close friends that live in your neighborhood") among neighbors.[24]

Dangerousness was measured from police records of crime incidence, from neighborhood residents' ratings of how much they feared for their safety and whether they had been victimized, and from independent raters' assessments of neighborhood safety based on the "virtual walk-through" using Google Street View.

© 2021 Reuben A et al. *JAMA Network Open.*

Data sources used

1. Geodemographic Data from Local Governments. We obtained information about the Index of Multiple Deprivation from the Department for Communities and Local Government. The Index is the official measure of relative deprivation for neighborhoods in England. Every small area in England is ranked from 1 (most deprived area) to 32,844 (least deprived area), these rankings are then converted into deciles. The Index of Multiple Deprivation is created based on 37 separate indicators, that are organized in seven domains of deprivation (Income Deprivation, Employment Deprivation; Education, Skills and Training Deprivation; Health Deprivation and Disability; Crime; Barriers to Housing and Services; and Living Environment Deprivation), and combined with appropriate weights to calculate the Index of Multiple Deprivation (IMD). Households were assigned a neighborhood IMD based on street address at the time of the age-5, age-7, age-10, and age-12 in-home visits using scores from the 2015 IMD. We analyzed the average IMD value across these four measurements.
2. Official Crime Data. We measured local area crime by mapping a 1-mile radius around each E-Risk Study family's home and tallying the total number of crimes that occurred in the area each month in 2011, when the participants were aged 17. Street-level crime data, including information on the type of crime, date of occurrence, and approximate location, were accessed online as part of an open data sharing effort about crime and policing in England and Wales (https://data.police.uk/) and geocoded to the home address of the Study members. An Application Program Interface was used to extract street-level crime data for each of the geospatial coordinates marking

© 2021 Reuben A et al. *JAMA Network Open.*

the family's home. For a full description see: https://data.police.uk/about/#location-anonymisation.

3. Google Street View Virtual Systematic Social Observation (SSO). The Google Street View SSO consisted of trained raters taking a "virtual walk" through the neighborhoods of the E-Risk families based on their street address at the time of the age-12 home visit. Raters then coded neighborhoods based on what they saw on that virtual walk. Street View is a freely available tool that generates panoramic street-level views using high definition images taken from camera-equipped cars. Signals from global positioning devices are used to accurately position images in the online maps. To avoid gaps in the imagery, adjacent cameras on the car take overlapping pictures and the images are then stitched together to create a continuous 360-degree image of the street. Images are then smoothed and re-projected onto a sphere to create the image displayed in Street View. To protect the privacy of individuals, face- and license-blurring technology is applied to ensure that people on the street and cars in the photographs cannot be identified. Google Street View came online in the United Kingdom in March 2009 and by March 2010, 94% of the E-Risk children's neighborhoods were available for viewing. The Google Street View SSO was completed by adapting SSO instruments for the virtual context and training raters to reliably code neighborhood features while taking a virtual walk down the street. We have reported full details of the Google Street View SSO method, inter-rater reliability and predictive validity of the measures elsewhere.[22] We analyzed Google Street View SSO measures of environmental decay and disorder and perceived dangerousness.

© 2021 Reuben A et al. *JAMA Network Open.*

4. Resident Surveys. A survey of residents living alongside E-Risk families was conducted when the children were 13-14 years of age to capture neighborhood-level social processes that cannot easily be captured via official records or direct observation. The sampling frame for the Neighborhood Survey was drawn using UK-Info Pro V13 http://www.192.com/products/. The survey responses were anonymous; no identifying information was collected. In Britain, a postcode area typically contains 15 households, with at most 100 households (e.g., a large apartment block). Therefore, survey respondents were typically living on the same street or within the same apartment block as the children in our study. Surveys were mailed to every household in the postcode registered to the electoral role, with the exception of the E-Risk family, resulting in 20,529 surveys being mailed to households to capture information on E-Risk families. On average, we received 5 (SD=3) completed surveys per neighborhood (range=0-18 respondents). We achieved at least 3 responses for 80% of target neighborhoods and at least 2 responses for 95% (resulting in a total of 5601 completed questionnaires). Survey responses were received for N=1,077 of the 1,116 families in the study. We analyzed survey measures of the following neighborhood-level social processes: fear of crime, direct victimization, neighborhood problems, and social disconnectedness.

Urbanicity

Urbanicity was derived from the UK Office for National Statitsic's (ONS) Rural-Urban Definition for Small Area Geographies (RUC2011) classifications.[25] The ONS classifications utilised 2011 census data, and were designed for application to small geostatistical units (e.g. Output Areas). Briefly, RUC2011 was created by laying a grid of

© 2021 Reuben A et al. *JAMA Network Open.*

hectare cells (100m^2) over England and Wales. Postcode addresses were assigned to cells, and residential densities were then calculated for increasing radii around each cell, providing each residential property with a density profile. This was combined with Output Area and contextual data, allowing each settlement to be assigned to one of ten urbanicity categories (Rural categories: sparse/non-sparse hamlets and isolated dwellings, sparse/non-sparse villages, sparse/nonsparse rural town and fringe; Urban categories: sparse/non-sparse city and town, and minor/major conurbations [conurbations are densely populated, large urban regions resulting from the expansion and coalescence of adjacent cities and towns]). ONS urbanicity scores were then assigned to every E-Risk family via the family's postcode when children were aged 5, 7, 10, 12 and 18. Given the low numbers within some rural categories, urbanicity was collapsed into three levels (1: "rural" = all rural categories [19.7% of participants at age 18]; 2: "intermediate" = urban cities and towns [48.4% of participants at age 18]; and 3: "urban" = minor/major conurbations [31.9% of participants at age 18]). E-Risk participants are nationally-representative in terms of ONS urbanicity classifications; 31.9% of E-Risk participants lived in urban settings at age 18 compared to 36.1% nationwide; 48.4% versus 45.0% lived in intermediate settings; and 19.7% versus 18.9% lived in rural settings.

© 2021 Reuben A et al. *JAMA Network Open.*

eAppendix 6. Comparison of participants with and without complete air pollution and psychopathology data.

2,039 participants (91.4% of original cohort; 1,071 [52.5%] female) had full air-pollution and psychopathology data. Participants with and without full data were not different on measures of childhood emotional ($t_{2,230}$=0.165, *P*=.869) and behavioral ($t_{2,230}$=0.409, *P*=.683) problems, smoking behavior ($t_{2,062}$=1.94, *P*=.053), or family psychiatric history ($t_{2,136}$=0.253, *P*=.800), although participants with full data were from families with slightly lower socioeconomic status (χ^2(1, N=2,232)=6.79, *P*=.034)).

© 2021 Reuben A et al. *JAMA Network Open.*

eReferences

1. Odgers CL, Caspi A, Russell MA, Sampson RJ, Arsenault L, Moffitt TE. Supportive parenting mediates widening neighborhood socioeconomic disparities in children's antisocial behavior from ages 5 to 12. Dev Psychopathol. 2012;24(3):705–21.

2. Moffitt TE, E-Risk Study Team. Teen-aged mothers in contemporary Britain. J Child Psychol Psychiatry. 2002;43(6):727–42.

3. Byun D, Schere KL. Review of the governing equations, computational algorithms, and other components of the models-3 Community Multiscale Air Quality (CMAQ) Modeling System. Appl Mech Rev. 2006;59(2):51-77. doi:10.1115/1.2128636

4. Beevers SD, Kitwiroon N, Williams ML, Carslaw DC. One way coupling of CMAQ and a road source dispersion model for fine scale air pollution predictions. Atmos Environ. 2012;59:47-58. doi:10.1016/j.atmosenv.2012.05.034

5. Schaefer JD, Moffitt TE, Arseneault L, et al. Adolescent victimization and early-adult psychopathology: approaching causal inference using a longitudinal twin study to rule out noncausal explanations. Clin Psychol Sci. 2018;6(3):352–71.

6. American Psychiatric Association. Diagnostic and Statistical Manual of Mental Disorders. 4th ed. Washington, DC: American Psychiatric Association; 1994.

7. Robins L, Cottler L, Bucholz K, Compton W. Diagnostic Interview Schedule for DSM-IV. St. Louis: Washington University School of Medicine; 1995.

8. Heatherton TF, Kozlowski LT, Frecker RC, Fagerström KO. The Fagerström Test for Nicotine Dependence: a revision of the Fagerström Tolerance Questionnaire. Br J Addict. 1991;86(9):1119–27.

9. American Psychiatric Association. Diagnostic and Statistical Manual of Mental Disorders. 5th ed. Washington, DC: American Psychiatric Association; 2013.

10. Agnew-Blais JC, Polanczyk GV, Danese A, Wertz J, Moffitt TE, Arseneault L. Evaluation of the persistence, remission, and emergence of Attention-Deficit/Hyperactivity Disorder in young adulthood. JAMA Psychiatry. 2016;73(7):713–20.

11. Morgan JF, Reid F, Lacey JH. The SCOFF questionnaire: assessment of a new screening tool for eating disorders. BMJ. 1999;319(7223):1467–8.

12. Polanczyk G, Moffitt TE, Arseneault L, et al. Etiological and clinical features of childhood psychotic symptoms: results from a birth cohort. Arch Gen Psychiatry. 2010;67(4):328–38.

© 2021 Reuben A et al. *JAMA Network Open.*

13. Loewy RL, Pearson R, Vinogradov S, Bearden CE, Cannon TD. Psychosis risk screening with the Prodromal Questionnaire--brief version (PQ-B). Schizophr Res. 2011;129(1):42–6.

14. Brunner M, Nagy G, Wilhelm O. A tutorial on hierarchically structured constructs. J Pers. 2012;80(4):796–846.

15. Rindskopf D, Rose T. Some theory and applications of Confirmatory Second-Order Factor Analysis. Multivar Behav Res. 1988;23(1):51–67.

16. Kotov R, Krueger RF, Watson D, Achenbach TM, Althoff RR, Bagby RM, et al. The Hierarchical Taxonomy of Psychopathology (HiTOP): a dimensional alternative to traditional nosologies. J Abnorm Psychol. 2017;126(4):454–77.

17. Muthén LK, Muthén BO. MPlus User's Guide. 8th ed. Los Angeles, CA: Muthén & Muthén; 2017.

18. Trzesniewski KH, Moffitt TE, Caspi A, Taylor A, Maughan B. Revisiting the association between reading achievement and antisocial behavior: new evidence of an environmental explanation from a twin study. Child Dev. 2006;77(1):72–88.

19. Milne BJ, Caspi A, Crump R, Poulton R, Rutter M, Sears MR, et al. The validity of the family history screen for assessing family history of mental disorders. Am J Med Genet Part B Neuropsychiatr Genet. 2009;150B(1):41–9.

20. Achenbach TM. Manual for the Teacher's Report Form and 1991 Profile. Department of Psychiatry, University of Vermont; 1991.

21. Achenbach TM. Manual for the Child Behavior Checklist and 1991 Profile. Department of Psychiatry, University of Vermont; 1991.

22. Odgers CL, Caspi A, Bates CJ, Sampson RJ, Moffitt TE. Systematic social observation of children's neighborhoods using Google Street View: a reliable and cost-effective method. J Child Psychol Psychiatry. 2012;53(10):1009–17.

23. Sampson RJ, Raudenbush SW, Earls F. Neighborhoods and violent crime: a multilevel study of collective efficacy. Science. 1997;277(5328):918–24.

24. Sampson RJ, Morenoff JD, Earls F. Beyond social capital: spatial dynamics of collective efficacy for children. Am Sociol Rev. 1999;64(5):633–60.

25. Bibby P, Brindley P. Urban and rural area definitions for policy purposes in England and Wales: Methodology (v1.0). London, UK: UK Office for National Statistics; 2013 p. 36.

© 2021 Reuben A et al. *JAMA Network Open.*
